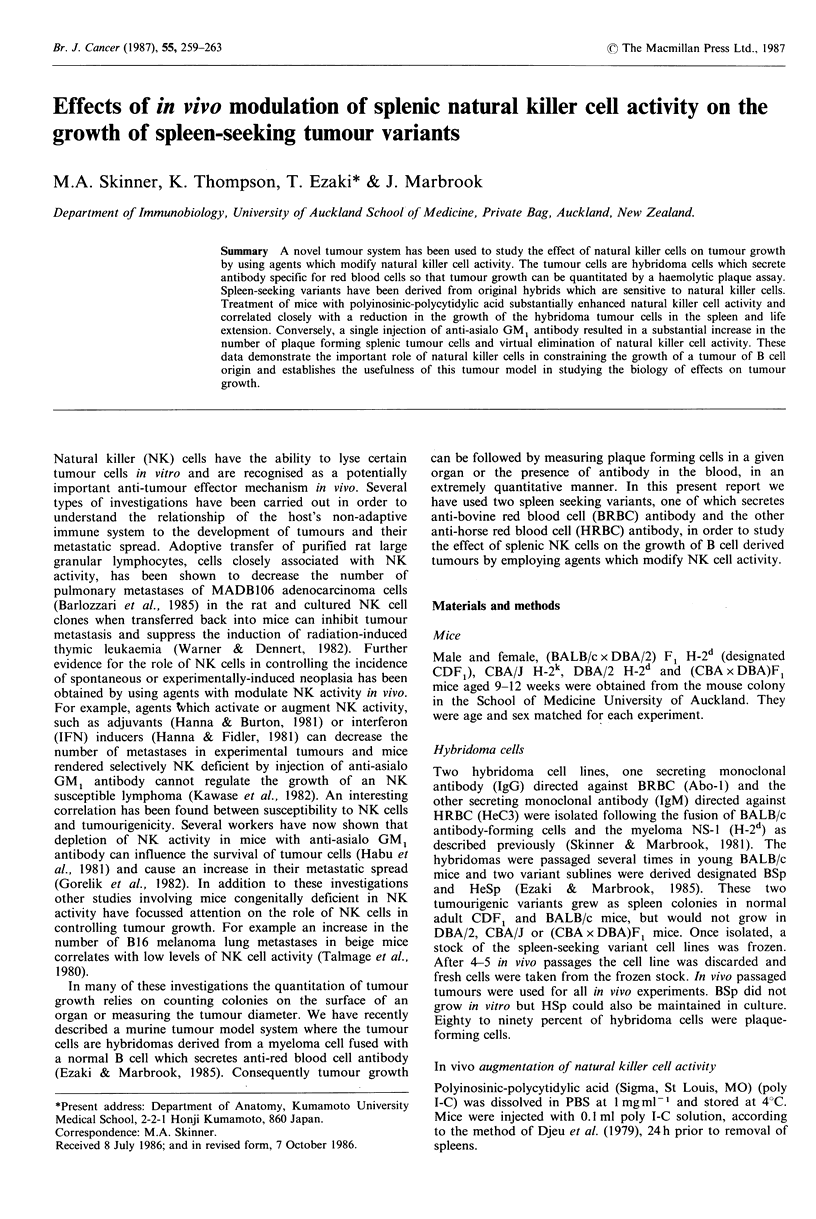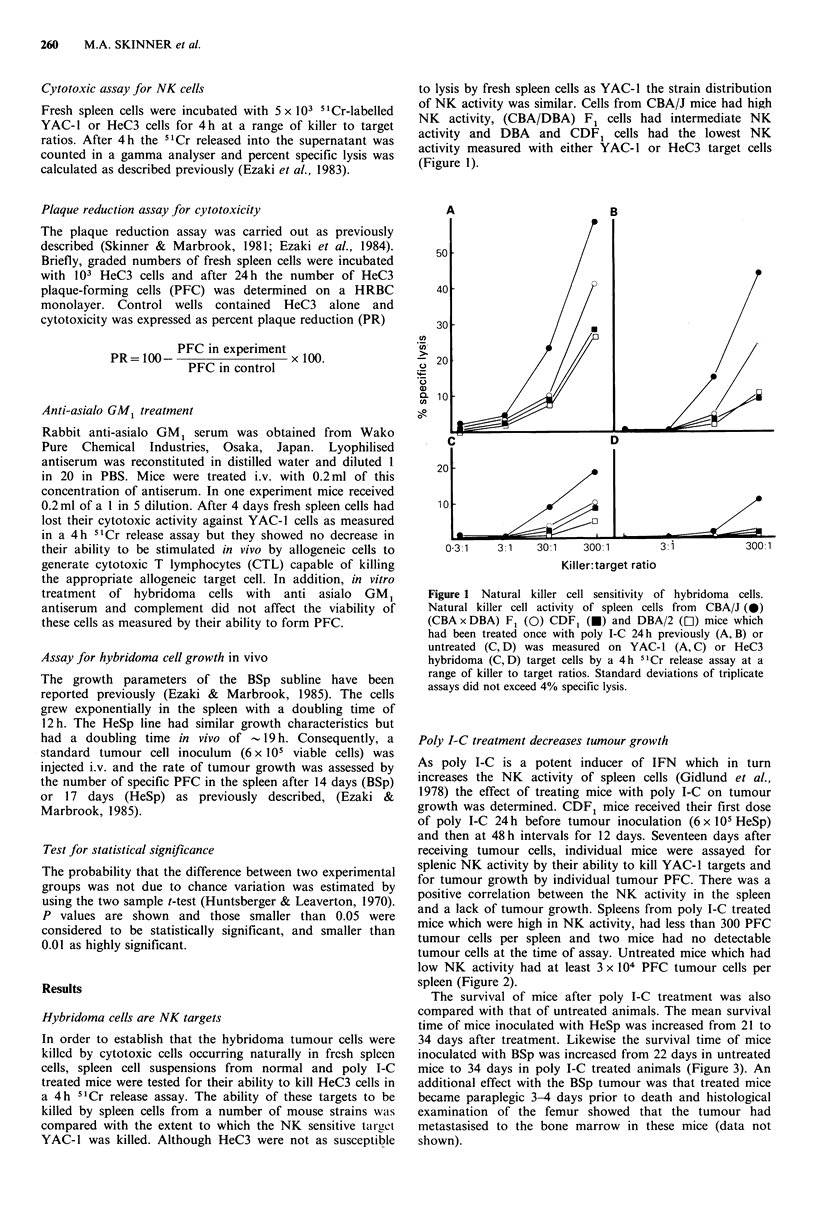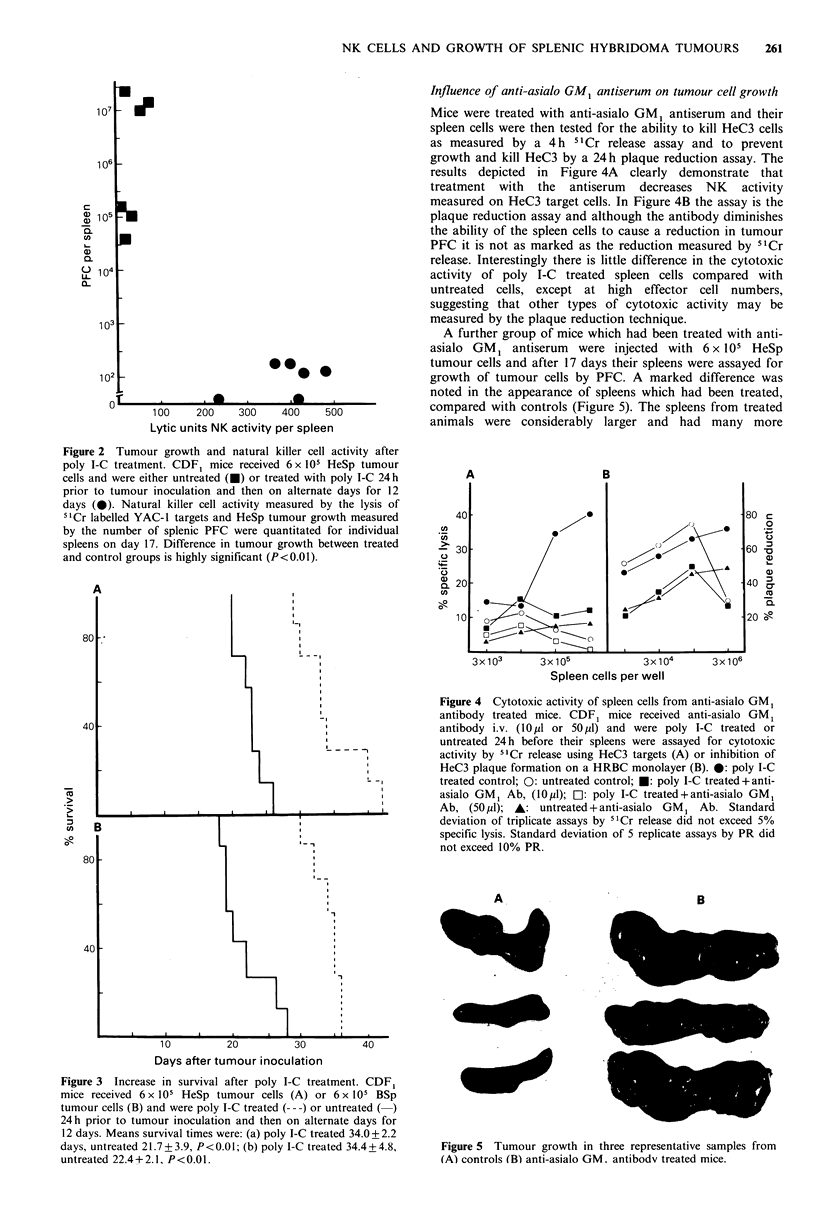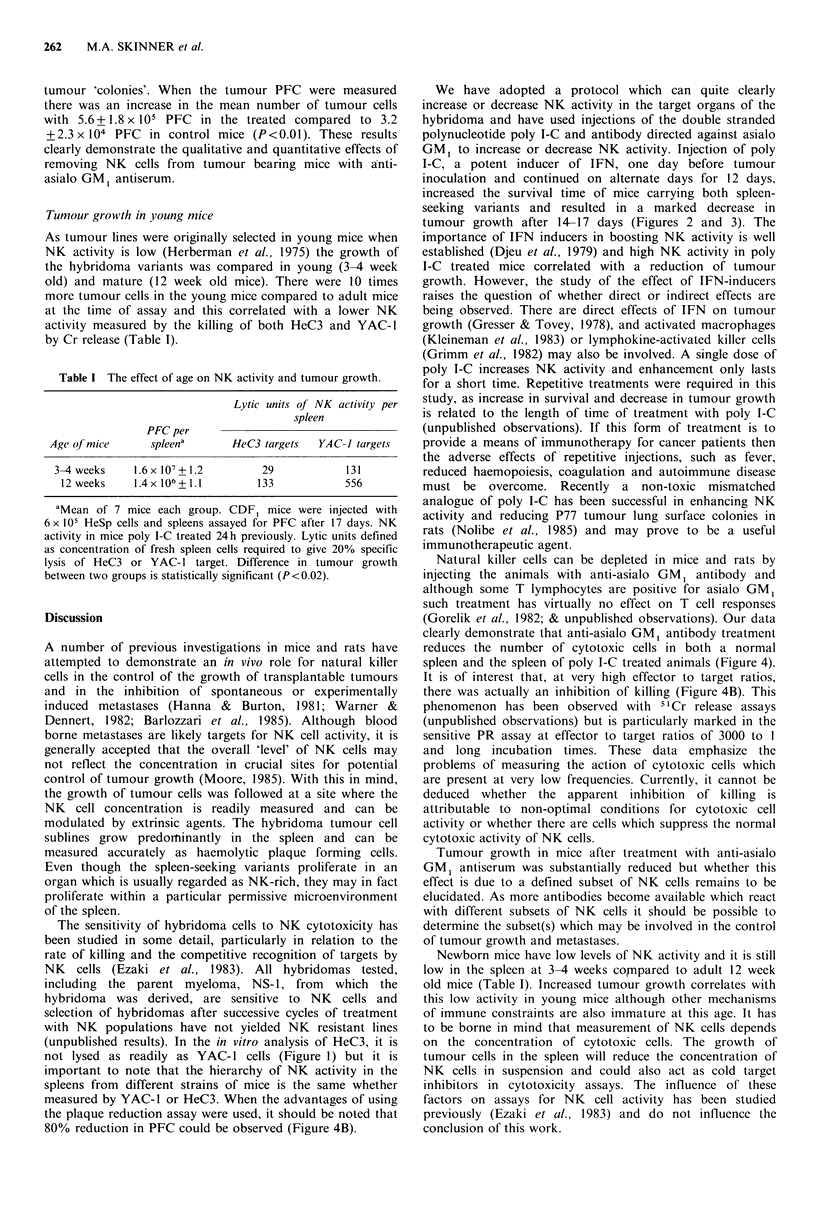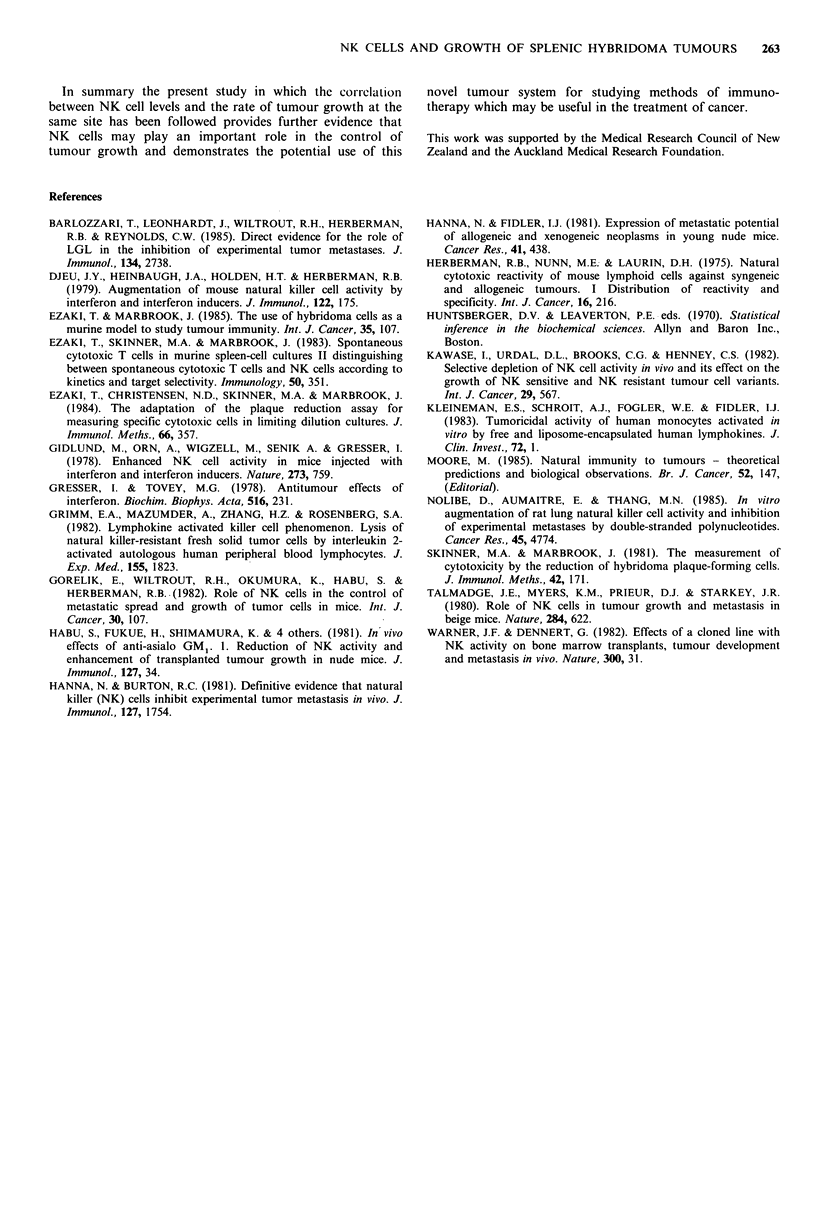# Effects of in vivo modulation of splenic natural killer cell activity on the growth of spleen-seeking tumour variants.

**DOI:** 10.1038/bjc.1987.50

**Published:** 1987-03

**Authors:** M. A. Skinner, K. Thompson, T. Ezaki, J. Marbrook

## Abstract

**Images:**


Br. J. Cancer (1987), 55, 259–263 © The Macmillan Press Ltd., 1987

### Effects of *in vivo* modulation of splenic natural killer cell activity on the growth of spleen-seeking tumour variants

M.A. Skinner, K. Thompson, T. Ezaki* & J. Marbrook

*Department of Immunobiology, University of Auckland School of Medicine, Private Bag, Auckland, New Zealand.*

**Summary** A novel tumour system has been used to study the effect of natural killer cells on tumour growth by using agents which modify natural killer cell activity. The tumour cells are hybridoma cells which secrete antibody specific for red blood cells so that tumour growth can be quantitated by a haemolytic plaque assay. Spleen-seeking variants have been derived from original hybrids which are sensitive to natural killer cells. Treatment of mice with polyinosinic-polycytidylic acid substantially enhanced natural killer cell activity and correlated closely with a reduction in the growth of the hybridoma tumour cells in the spleen and life extension. Conversely, a single injection of anti-asialo $GM_1$ antibody resulted in a substantial increase in the number of plaque forming splenic tumour cells and virtual elimination of natural killer cell activity. These data demonstrate the important role of natural killer cells in constraining the growth of a tumour of B cell origin and establishes the usefulness of this tumour model in studying the biology of effects on tumour growth.

Natural killer (NK) cells have the ability to lyse certain tumour cells *in vitro* and are recognised as a potentially important anti-tumour effector mechanism *in vivo*. Several types of investigations have been carried out in order to understand the relationship of the host's non-adaptive immune system to the development of tumours and their metastatic spread. Adoptive transfer of purified rat large granular lymphocytes, cells closely associated with NK activity, has been shown to decrease the number of pulmonary metastases of MADB106 adenocarcinoma cells (Barlozzari *et al.*, 1985) in the rat and cultured NK cell clones when transferred back into mice can inhibit tumour metastasis and suppress the induction of radiation-induced thymic leukaemia (Warner & Dennert, 1982). Further evidence for the role of NK cells in controlling the incidence of spontaneous or experimentally-induced neoplasia has been obtained by using agents with modulate NK activity *in vivo*. For example, agents which activate or augment NK activity, such as adjuvants (Hanna & Burton, 1981) or interferon (IFN) inducers (Hanna & Fidler, 1981) can decrease the number of metastases in experimental tumours and mice rendered selectively NK deficient by injection of anti-asialo $GM_1$ antibody cannot regulate the growth of an NK susceptible lymphoma (Kawase *et al.*, 1982). An interesting correlation has been found between susceptibility to NK cells and tumourigenicity. Several workers have now shown that depletion of NK activity in mice with anti-asialo $GM_1$ antibody can influence the survival of tumour cells (Habu *et al.*, 1981) and cause an increase in their metastatic spread (Gorelik *et al.*, 1982). In addition to these investigations other studies involving mice congenitally deficient in NK activity have focussed attention on the role of NK cells in controlling tumour growth. For example an increase in the number of B16 melanoma lung metastases in beige mice correlates with low levels of NK cell activity (Talmage *et al.*, 1980).

In many of these investigations the quantitation of tumour growth relies on counting colonies on the surface of an organ or measuring the tumour diameter. We have recently described a murine tumour model system where the tumour cells are hybridomas derived from a myeloma cell fused with a normal B cell which secretes anti-red blood cell antibody (Ezaki & Marbrook, 1985). Consequently tumour growth can be followed by measuring plaque forming cells in a given organ or the presence of antibody in the blood, in an extremely quantitative manner. In this present report we have used two spleen seeking variants, one of which secretes anti-bovine red blood cell (BRBC) antibody and the other anti-horse red blood cell (HRBC) antibody, in order to study the effect of splenic NK cells on the growth of B cell derived tumours by employing agents which modify NK cell activity.

*Present address: Department of Anatomy, Kumamoto University Medical School, 2-2-1 Honji Kumamoto, 860 Japan.
Correspondence: M.A. Skinner.
Received 8 July 1986; and in revised form, 7 October 1986.

### Materials and methods

### *Mice*

Male and female, (BALB/c × DBA/2) $F_1$ H-$2^d$ (designated $CDF_1$), CBA/J H-$2^k$, DBA/2 H-$2^d$ and (CBA × DBA)$F_1$ mice aged 9–12 weeks were obtained from the mouse colony in the School of Medicine University of Auckland. They were age and sex matched for each experiment.

### *Hybridoma cells*

Two hybridoma cell lines, one secreting monoclonal antibody (IgG) directed against BRBC (Abo-1) and the other secreting monoclonal antibody (IgM) directed against HRBC (HeC3) were isolated following the fusion of BALB/c antibody-forming cells and the myeloma NS-1 (H-$2^d$) as described previously (Skinner & Marbrook, 1981). The hybridomas were passaged several times in young BALB/c mice and two variant sublines were derived designated BSp and HeSp (Ezaki & Marbrook, 1985). These two tumourigenic variants grew as spleen colonies in normal adult $CDF_1$ and BALB/c mice, but would not grow in DBA/2, CBA/J or (CBA × DBA)$F_1$ mice. Once isolated, a stock of the spleen-seeking variant cell lines was frozen. After 4–5 *in vivo* passages the cell line was discarded and fresh cells were taken from the frozen stock. *In vivo* passaged tumours were used for all *in vivo* experiments. BSp did not grow *in vitro* but HSp could also be maintained in culture. Eighty to ninety percent of hybridoma cells were plaque-forming cells.

### In vivo *augmentation of natural killer cell activity*

Polyinosinic-polycytidylic acid (Sigma, St Louis, MO) (poly I-C) was dissolved in PBS at $1\,mg\,ml^{-1}$ and stored at 4°C. Mice were injected with 0.1 ml poly I-C solution, according to the method of Djeu *et al.* (1979), 24 h prior to removal of spleens.

*Cytotoxic assay for NK cells*

Fresh spleen cells were incubated with $5 \times 10^3$ $^{51}$Cr-labelled YAC-1 or HeC3 cells for 4 h at a range of killer to target ratios. After 4 h the $^{51}$Cr released into the supernatant was counted in a gamma analyser and percent specific lysis was calculated as described previously (Ezaki *et al.*, 1983).

*Plaque reduction assay for cytotoxicity*

The plaque reduction assay was carried out as previously described (Skinner & Marbrook, 1981; Ezaki *et al.*, 1984). Briefly, graded numbers of fresh spleen cells were incubated with $10^3$ HeC3 cells and after 24 h the number of HeC3 plaque-forming cells (PFC) was determined on a HRBC monolayer. Control wells contained HeC3 alone and cytotoxicity was expressed as percent plaque reduction (PR)

$$PR = 100 - \frac{\text{PFC in experiment}}{\text{PFC in control}} \times 100.$$

*Anti-asialo $GM_1$ treatment*

Rabbit anti-asialo $GM_1$ serum was obtained from Wako Pure Chemical Industries, Osaka, Japan. Lyophilised antiserum was reconstituted in distilled water and diluted 1 in 20 in PBS. Mice were treated i.v. with 0.2 ml of this concentration of antiserum. In one experiment mice received 0.2 ml of a 1 in 5 dilution. After 4 days fresh spleen cells had lost their cytotoxic activity against YAC-1 cells as measured in a 4 h $^{51}$Cr release assay but they showed no decrease in their ability to be stimulated *in vivo* by allogeneic cells to generate cytotoxic T lymphocytes (CTL) capable of killing the appropriate allogeneic target cell. In addition, *in vitro* treatment of hybridoma cells with anti asialo $GM_1$ antiserum and complement did not affect the viability of these cells as measured by their ability to form PFC.

*Assay for hybridoma cell growth* in vivo

The growth parameters of the BSp subline have been reported previously (Ezaki & Marbrook, 1985). The cells grew exponentially in the spleen with a doubling time of 12 h. The HeSp line had similar growth characteristics but had a doubling time *in vivo* of ~19 h. Consequently, a standard tumour cell inoculum ($6 \times 10^5$ viable cells) was injected i.v. and the rate of tumour growth was assessed by the number of specific PFC in the spleen after 14 days (BSp) or 17 days (HeSp) as previously described, (Ezaki & Marbrook, 1985).

*Test for statistical significance*

The probability that the difference between two experimental groups was not due to chance variation was estimated by using the two sample *t*-test (Huntsberger & Leaverton, 1970). *P* values are shown and those smaller than 0.05 were considered to be statistically significant, and smaller than 0.01 as highly significant.

### Results

*Hybridoma cells are NK targets*

In order to establish that the hybridoma tumour cells were killed by cytotoxic cells occurring naturally in fresh spleen cells, spleen cell suspensions from normal and poly I-C treated mice were tested for their ability to kill HeC3 cells in a 4 h $^{51}$Cr release assay. The ability of these targets to be killed by spleen cells from a number of mouse strains was compared with the extent to which the NK sensitive target YAC-1 was killed. Although HeC3 were not as susceptible to lysis by fresh spleen cells as YAC-1 the strain distribution of NK activity was similar. Cells from CBA/J mice had high NK activity, (CBA/DBA) $F_1$ cells had intermediate NK activity and DBA and $CDF_1$ cells had the lowest NK activity measured with either YAC-1 or HeC3 target cells (Figure 1).

**Figure 1** Natural killer cell sensitivity of hybridoma cells. Natural killer cell activity of spleen cells from CBA/J (●) (CBA × DBA) $F_1$ (○) $CDF_1$ (■) and DBA/2 (□) mice which had been treated once with poly I-C 24 h previously (A, B) or untreated (C, D) was measured on YAC-1 (A, C) or HeC3 hybridoma (C, D) target cells by a 4 h $^{51}$Cr release assay at a range of killer to target ratios. Standard deviations of triplicate assays did not exceed 4% specific lysis.

*Poly I-C treatment decreases tumour growth*

As poly I-C is a potent inducer of IFN which in turn increases the NK activity of spleen cells (Gidlund *et al.*, 1978) the effect of treating mice with poly I-C on tumour growth was determined. $CDF_1$ mice received their first dose of poly I-C 24 h before tumour inoculation ($6 \times 10^5$ HeSp) and then at 48 h intervals for 12 days. Seventeen days after receiving tumour cells, individual mice were assayed for splenic NK activity by their ability to kill YAC-1 targets and for tumour growth by individual tumour PFC. There was a positive correlation between the NK activity in the spleen and a lack of tumour growth. Spleens from poly I-C treated mice which were high in NK activity, had less than 300 PFC tumour cells per spleen and two mice had no detectable tumour cells at the time of assay. Untreated mice which had low NK activity had at least $3 \times 10^4$ PFC tumour cells per spleen (Figure 2).

The survival of mice after poly I-C treatment was also compared with that of untreated animals. The mean survival time of mice inoculated with HeSp was increased from 21 to 34 days after treatment. Likewise the survival time of mice inoculated with BSp was increased from 22 days in untreated mice to 34 days in poly I-C treated animals (Figure 3). An additional effect with the BSp tumour was that treated mice became paraplegic 3–4 days prior to death and histological examination of the femur showed that the tumour had metastasised to the bone marrow in these mice (data not shown).

**Figure 2** Tumour growth and natural killer cell activity after poly I-C treatment. $CDF_1$ mice received $6\times10^5$ HeSp tumour cells and were either untreated (■) or treated with poly I-C 24 h prior to tumour inoculation and then on alternate days for 12 days (●). Natural killer cell activity measured by the lysis of $^{51}Cr$ labelled YAC-1 targets and HeSp tumour growth measured by the number of splenic PFC were quantitated for individual spleens on day 17. Difference in tumour growth between treated and control groups is highly significant ($P<0.01$).

**Figure 3** Increase in survival after poly I-C treatment. $CDF_1$ mice received $6\times10^5$ HeSp tumour cells (A) or $6\times10^5$ BSp tumour cells (B) and were poly I-C treated (- - -) or untreated (—) 24 h prior to tumour inoculation and then on alternate days for 12 days. Means survival times were: (a) poly I-C treated $34.0\pm2.2$ days, untreated $21.7\pm3.9$, $P<0.01$; (b) poly I-C treated $34.4\pm4.8$, untreated $22.4+2.1$, $P<0.01$.

*Influence of anti-asialo $GM_1$ antiserum on tumour cell growth*

Mice were treated with anti-asialo $GM_1$ antiserum and their spleen cells were then tested for the ability to kill HeC3 cells as measured by a 4 h $^{51}Cr$ release assay and to prevent growth and kill HeC3 by a 24 h plaque reduction assay. The results depicted in Figure 4A clearly demonstrate that treatment with the antiserum decreases NK activity measured on HeC3 target cells. In Figure 4B the assay is the plaque reduction assay and although the antibody diminishes the ability of the spleen cells to cause a reduction in tumour PFC it is not as marked as the reduction measured by $^{51}Cr$ release. Interestingly there is little difference in the cytotoxic activity of poly I-C treated spleen cells compared with untreated cells, except at high effector cell numbers, suggesting that other types of cytotoxic activity may be measured by the plaque reduction technique.

A further group of mice which had been treated with anti-asialo $GM_1$ antiserum were injected with $6\times10^5$ HeSp tumour cells and after 17 days their spleens were assayed for growth of tumour cells by PFC. A marked difference was noted in the appearance of spleens which had been treated, compared with controls (Figure 5). The spleens from treated animals were considerably larger and had many more

**Figure 4** Cytotoxic activity of spleen cells from anti-asialo $GM_1$ antibody treated mice. $CDF_1$ mice received anti-asialo $GM_1$ antibody i.v. (10 μl or 50 μl) and were poly I-C treated or untreated 24 h before their spleens were assayed for cytotoxic activity by $^{51}Cr$ release using HeC3 targets (A) or inhibition of HeC3 plaque formation on a HRBC monolayer (B). ●: poly I-C treated control; ○: untreated control; ■: poly I-C treated + anti-asialo $GM_1$ Ab, (10 μl); □: poly I-C treated + anti-asialo $GM_1$ Ab, (50 μl); ▲: untreated + anti-asialo $GM_1$ Ab. Standard deviation of triplicate assays by $^{51}Cr$ release did not exceed 5% specific lysis. Standard deviation of 5 replicate assays by PR did not exceed 10% PR.

**Figure 5** Tumour growth in three representative samples from (A) controls (B) anti-asialo GM, antibody treated mice.

tumour 'colonies'. When the tumour PFC were measured there was an increase in the mean number of tumour cells with $5.6 \pm 1.8 \times 10^5$ PFC in the treated compared to $3.2 \pm 2.3 \times 10^4$ PFC in control mice ($P<0.01$). These results clearly demonstrate the qualitative and quantitative effects of removing NK cells from tumour bearing mice with anti-asialo $GM_1$ antiserum.

*Tumour growth in young mice*

As tumour lines were originally selected in young mice when NK activity is low (Herberman *et al.*, 1975) the growth of the hybridoma variants was compared in young (3–4 week old) and mature (12 week old mice). There were 10 times more tumour cells in the young mice compared to adult mice at the time of assay and this correlated with a lower NK activity measured by the killing of both HeC3 and YAC-1 by Cr release (Table I).

**Table I** The effect of age on NK activity and tumour growth.

| *Age of mice* | *PFC per spleen*[a] | *Lytic units of NK activity per spleen* | |
|---|---|---|---|
| | | *HeC3 targets* | *YAC-1 targets* |
| 3–4 weeks | $1.6 \times 10^7 \pm 1.2$ | 29 | 131 |
| 12 weeks | $1.4 \times 10^6 \pm 1.1$ | 133 | 556 |

[a]Mean of 7 mice each group. $CDF_1$ mice were injected with $6 \times 10^5$ HeSp cells and spleens assayed for PFC after 17 days. NK activity in mice poly I-C treated 24 h previously. Lytic units defined as concentration of fresh spleen cells required to give 20% specific lysis of HeC3 or YAC-1 target. Difference in tumour growth between two groups is statistically significant ($P<0.02$).

### Discussion

A number of previous investigations in mice and rats have attempted to demonstrate an *in vivo* role for natural killer cells in the control of the growth of transplantable tumours and in the inhibition of spontaneous or experimentally induced metastases (Hanna & Burton, 1981; Warner & Dennert, 1982; Barlozzari *et al.*, 1985). Although blood borne metastases are likely targets for NK cell activity, it is generally accepted that the overall 'level' of NK cells may not reflect the concentration in crucial sites for potential control of tumour growth (Moore, 1985). With this in mind, the growth of tumour cells was followed at a site where the NK cell concentration is readily measured and can be modulated by extrinsic agents. The hybridoma tumour cell sublines grow predominantly in the spleen and can be measured accurately as haemolytic plaque forming cells. Even though the spleen-seeking variants proliferate in an organ which is usually regarded as NK-rich, they may in fact proliferate within a particular permissive microenvironment of the spleen.

The sensitivity of hybridoma cells to NK cytotoxicity has been studied in some detail, particularly in relation to the rate of killing and the competitive recognition of targets by NK cells (Ezaki *et al.*, 1983). All hybridomas tested, including the parent myeloma, NS-1, from which the hybridoma was derived, are sensitive to NK cells and selection of hybridomas after successive cycles of treatment with NK populations have not yielded NK resistant lines (unpublished results). In the *in vitro* analysis of HeC3, it is not lysed as readily as YAC-1 cells (Figure 1) but it is important to note that the hierarchy of NK activity in the spleens from different strains of mice is the same whether measured by YAC-1 or HeC3. When the advantages of using the plaque reduction assay were used, it should be noted that 80% reduction in PFC could be observed (Figure 4B).

We have adopted a protocol which can quite clearly increase or decrease NK activity in the target organs of the hybridoma and have used injections of the double stranded polynucleotide poly I-C and antibody directed against asialo $GM_1$ to increase or decrease NK activity. Injection of poly I-C, a potent inducer of IFN, one day before tumour inoculation and continued on alternate days for 12 days, increased the survival time of mice carrying both spleen-seeking variants and resulted in a marked decrease in tumour growth after 14–17 days (Figures 2 and 3). The importance of IFN inducers in boosting NK activity is well established (Djeu *et al.*, 1979) and high NK activity in poly I-C treated mice correlated with a reduction of tumour growth. However, the study of the effect of IFN-inducers raises the question of whether direct or indirect effects are being observed. There are direct effects of IFN on tumour growth (Gresser & Tovey, 1978), and activated macrophages (Kleineman *et al.*, 1983) or lymphokine-activated killer cells (Grimm *et al.*, 1982) may also be involved. A single dose of poly I-C increases NK activity and enhancement only lasts for a short time. Repetitive treatments were required in this study, as increase in survival and decrease in tumour growth is related to the length of time of treatment with poly I-C (unpublished observations). If this form of treatment is to provide a means of immunotherapy for cancer patients then the adverse effects of repetitive injections, such as fever, reduced haemopoiesis, coagulation and autoimmune disease must be overcome. Recently a non-toxic mismatched analogue of poly I-C has been successful in enhancing NK activity and reducing P77 tumour lung surface colonies in rats (Nolibe *et al.*, 1985) and may prove to be a useful immunotherapeutic agent.

Natural killer cells can be depleted in mice and rats by injecting the animals with anti-asialo $GM_1$ antibody and although some T lymphocytes are positive for asialo $GM_1$ such treatment has virtually no effect on T cell responses (Gorelik *et al.*, 1982; & unpublished observations). Our data clearly demonstrate that anti-asialo $GM_1$ antibody treatment reduces the number of cytotoxic cells in both a normal spleen and the spleen of poly I-C treated animals (Figure 4). It is of interest that, at very high effector to target ratios, there was actually an inhibition of killing (Figure 4B). This phenomenon has been observed with $^{51}Cr$ release assays (unpublished observations) but is particularly marked in the sensitive PR assay at effector to target ratios of 3000 to 1 and long incubation times. These data emphasize the problems of measuring the action of cytotoxic cells which are present at very low frequencies. Currently, it cannot be deduced whether the apparent inhibition of killing is attributable to non-optimal conditions for cytotoxic cell activity or whether there are cells which suppress the normal cytotoxic activity of NK cells.

Tumour growth in mice after treatment with anti-asialo $GM_1$ antiserum was substantially reduced but whether this effect is due to a defined subset of NK cells remains to be elucidated. As more antibodies become available which react with different subsets of NK cells it should be possible to determine the subset(s) which may be involved in the control of tumour growth and metastases.

Newborn mice have low levels of NK activity and it is still low in the spleen at 3–4 weeks compared to adult 12 week old mice (Table I). Increased tumour growth correlates with this low activity in young mice although other mechanisms of immune constraints are also immature at this age. It has to be borne in mind that measurement of NK cells depends on the concentration of cytotoxic cells. The growth of tumour cells in the spleen will reduce the concentration of NK cells in suspension and could also act as cold target inhibitors in cytotoxicity assays. The influence of these factors on assays for NK cell activity has been studied previously (Ezaki *et al.*, 1983) and do not influence the conclusion of this work.

In summary the present study in which the correlation between NK cell levels and the rate of tumour growth at the same site has been followed provides further evidence that NK cells may play an important role in the control of tumour growth and demonstrates the potential use of this novel tumour system for studying methods of immunotherapy which may be useful in the treatment of cancer.

This work was supported by the Medical Research Council of New Zealand and the Auckland Medical Research Foundation.

### References

BARLOZZARI, T., LEONHARDT, J., WILTROUT, R.H., HERBERMAN, R.B. & REYNOLDS, C.W. (1985). Direct evidence for the role of LGL in the inhibition of experimental tumor metastases. *J. Immunol.*, **134**, 2738.

DJEU, J.Y., HEINBAUGH, J.A., HOLDEN, H.T. & HERBERMAN, R.B. (1979). Augmentation of mouse natural killer cell activity by interferon and interferon inducers. *J. Immunol.*, **122**, 175.

EZAKI, T. & MARBROOK, J. (1985). The use of hybridoma cells as a murine model to study tumour immunity. *Int. J. Cancer*, **35**, 107.

EZAKI, T., SKINNER, M.A. & MARBROOK, J. (1983). Spontaneous cytotoxic T cells in murine spleen-cell cultures II distinguishing between spontaneous cytotoxic T cells and NK cells according to kinetics and target selectivity. *Immunology*, **50**, 351.

EZAKI, T., CHRISTENSEN, N.D., SKINNER, M.A. & MARBROOK, J. (1984). The adaptation of the plaque reduction assay for measuring specific cytotoxic cells in limiting dilution cultures. *J. Immunol. Meths.*, **66**, 357.

GIDLUND, M., ORN, A., WIGZELL, M., SENIK A. & GRESSER, I. (1978). Enhanced NK cell activity in mice injected with interferon and interferon inducers. *Nature*, **273**, 759.

GRESSER, I. & TOVEY, M.G. (1978). Antitumour effects of interferon. *Biochim. Biophys. Acta*, **516**, 231.

GRIMM, E.A., MAZUMDER, A., ZHANG, H.Z. & ROSENBERG, S.A. (1982). Lymphokine activated killer cell phenomenon. Lysis of natural killer-resistant fresh solid tumor cells by interleukin 2-activated autologous human peripheral blood lymphocytes. *J. Exp. Med.*, **155**, 1823.

GORELIK, E., WILTROUT, R.H., OKUMURA, K., HABU, S. & HERBERMAN, R.B. (1982). Role of NK cells in the control of metastatic spread and growth of tumor cells in mice. *Int. J. Cancer*, **30**, 107.

HABU, S., FUKUE, H., SHIMAMURA, K. & 4 others. (1981). *In vivo* effects of anti-asialo $GM_1$. 1. Reduction of NK activity and enhancement of transplanted tumour growth in nude mice. *J. Immunol.*, **127**, 34.

HANNA, N. & BURTON, R.C. (1981). Definitive evidence that natural killer (NK) cells inhibit experimental tumor metastasis *in vivo*. *J. Immunol.*, **127**, 1754.

HANNA, N. & FIDLER, I.J. (1981). Expression of metastatic potential of allogeneic and xenogeneic neoplasms in young nude mice. *Cancer Res.*, **41**, 438.

HERBERMAN, R.B., NUNN, M.E. & LAURIN, D.H. (1975). Natural cytotoxic reactivity of mouse lymphoid cells against syngeneic and allogeneic tumours. I Distribution of reactivity and specificity. *Int. J. Cancer*, **16**, 216.

HUNTSBERGER, D.V. & LEAVERTON, P.E. eds. (1970). *Statistical inference in the biochemical sciences.* Allyn and Baron Inc., Boston.

KAWASE, I., URDAL, D.L., BROOKS, C.G. & HENNEY, C.S. (1982). Selective depletion of NK cell activity *in vivo* and its effect on the growth of NK sensitive and NK resistant tumour cell variants. *Int. J. Cancer*, **29**, 567.

KLEINEMAN, E.S., SCHROIT, A.J., FOGLER, W.E. & FIDLER, I.J. (1983). Tumoricidal activity of human monocytes activated *in vitro* by free and liposome-encapsulated human lymphokines. *J. Clin. Invest.*, **72**, 1.

MOORE, M. (1985). Natural immunity to tumours – theoretical predictions and biological observations. *Br. J. Cancer*, **52**, 147, (*Editorial*).

NOLIBE, D., AUMAITRE, E. & THANG, M.N. (1985). *In vitro* augmentation of rat lung natural killer cell activity and inhibition of experimental metastases by double-stranded polynucleotides. *Cancer Res.*, **45**, 4774.

SKINNER, M.A. & MARBROOK, J. (1981). The measurement of cytotoxicity by the reduction of hybridoma plaque-forming cells. *J. Immunol. Meths.*, **42**, 171.

TALMADGE, J.E., MYERS, K.M., PRIEUR, D.J. & STARKEY, J.R. (1980). Role of NK cells in tumour growth and metastasis in beige mice. *Nature*, **284**, 622.

WARNER, J.F. & DENNERT, G. (1982). Effects of a cloned line with NK activity on bone marrow transplants, tumour development and metastasis *in vivo*. *Nature*, **300**, 31.